# Self-Propulsion Strategies for Artificial Cell-Like Compartments

**DOI:** 10.3390/nano9121680

**Published:** 2019-11-25

**Authors:** Ibon Santiago, Friedrich C. Simmel

**Affiliations:** Physics Department, Technical University of Munich, Am Coulombwall 4a, 85748 Garching, Germany

**Keywords:** active matter, artificial cells, synthetic biology, micro/nano motors, self-propulsion

## Abstract

Reconstitution of life-like properties in artificial cells is a current research frontier in synthetic biology. Mimicking metabolism, growth, and sensing are active areas of investigation; however, achieving motility and directional taxis are also challenging in the context of artificial cells. To tackle this problem, recent progress has been made that leverages the tools of active matter physics in synthetic biology. This review surveys the most significant achievements in designing motile cell-like compartments. In this context, strategies for self-propulsion are summarized, including, compartmentalization of catalytically active particles, phoretic propulsion of vesicles and emulsion droplet motion driven by Marangoni flows. This work showcases how the realization of motile protocells may impact biomedical engineering while also aiming at answering fundamental questions in locomotion of prebiotic cells.

## 1. Introduction

Recreating cellular functions within cell-like compartments represents a central objective of artificial life research. Bottom-up synthetic biology aims at generating simpler life-like entities from molecular components, which can be predicted and controlled with high precision [1]. These synthetic cells will help determine the minimal set of compounds and processes required to sustain life, while holding great potential to produce smart therapeutics and new biomaterials, such as artificial tissues [2].

Compartmentalization is a defining property of cells. Compartments create physical boundaries that allow cells to carry out specific biological processes, such as metabolism, growth, and replication. Most notably, the cell membrane separates and protects the cell from its surroundings, creating a chemical micro-environment with controlled molecular composition and exchange with the environment. The goal of constructing an artificial cell from basic molecular components has inspired many researchers to create synthetic cell-like compartments [3]. Recent progress has enabled the construction of biomimetic colloidal entities that can serve as compartments. Such synthetic compartments are made of naturally occurring molecules, like phospholipids or peptides, as well as synthetic molecules like block co-polymers, and other surfactants [4]. They are used to create protocell structures (precursors to modern cells formed from abiotic components) ranging from lipid and polymer vesicles to emulsion droplets and membrane-less compartments (coacervates). Aqueous droplets covered by a continuous membranous bilayer (made of lipids or polymers) in a bulk aqueous phase form stable vesicles which can be used to encapsulate molecules and mimic certain properties of the cell membrane. Droplets formed by emulsification of immiscible components (e.g., water-in-oil) or by coacervation (liquid–liquid phase separation of charged macromolecules) can also be used as cell-like compartments, but lacking the complexity of a membranous bilayer.

A further step towards assembling more versatile artificial cells would involve the capacity of locomotion. Implementing directed motion is a first step for applications where vesicles can deliver drugs to targeted cells or perform specific tasks in biological environments. However, the realization of self-propulsion within cell-like compartments remains challenging. Self-propelled particles can convert stored or ambient free energy into systematic movement [5]. While a wealth of propulsion strategies for inorganic colloidal microparticles (e.g., Janus microswimmers [6]) had been previously put forward by the active matter physics community [7], only recently there has been a growing interest in introducing self-propulsion in soft and organic synthetic compartments like polymersomes [8,9].

The focus of this work is how biomimetic cells can be engineered to exhibit locomotion and to summarize the recent experimental progress. Two distinct propulsion strategies are studied that have been realized in several systems, including nanoscale membranous vesicles (e.g., liposomes and polymersomes), and emulsion droplets. These are the first attempts to create self-propelled cell-like compartments, which will play a role in the development of more realistic motile cell models, including active double emulsions and cell-sized vesicles.

## 2. Propulsion Mechanisms

The field of active matter physics has flourished in the past decades with numerous realizations of colloidal self-propelled particles [10]. Among the many propulsion mechanisms, two strategies stand out that are directly applicable to cell-like compartments: phoresis and the Marangoni effect. The former takes advantage of interactions between the particle surface and the surrounding medium to generate propulsion. The latter is based on surface tension gradients that induce a fluid flow, known as Marangoni flow, resulting in propulsion.

### 2.1. Phoretic Motion: Diffusiophoresis

**Figure 1:**
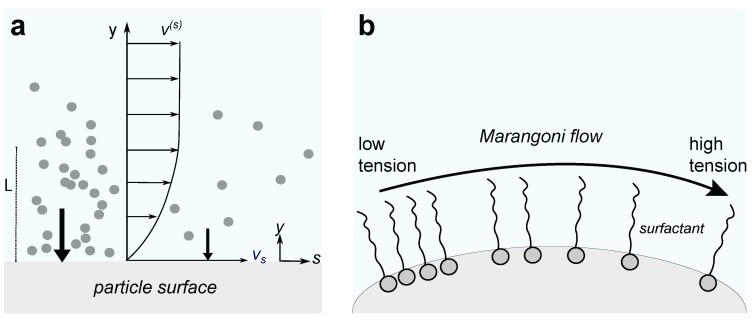
(**a**) Diffusiophoresis for a neutral solute (grey circles) attracted to the surface. A chemical reaction generates a concentration gradient with higher solute concentration on the left side (L indicates the thickness of the boundary layer interface) ‘pushing’ the fluid against the surface. A pressure gradient results in the interfacial layer, which creates a flow opposite to the concentration gradient, from high concentration to low, indicated by thick and thin arrows, respectively. An apparent slip velocity builds up around the particle with its asymptotic value reached at $v_{s}\left(s,y=L\right)=v^{\left(s\right)}$; (**b**) Marangoni flow: variation in surface tension on a surfactant-laden droplet interface leading to a fluid flow from low to high surface tension areas.

Phoresis refers to a type of transport that arises by external fields interacting with the interfacial layer of particles [11]. These interactions create an active flow that leads to directed motion. The phoretic force may be, for example, due to an external temperature gradient (thermophoresis) or a chemical gradient (diffusiophoresis). When the gradient is generated by the particle itself (self-phoresis), it gives rise to self-generated forces, resulting in self-propulsion. For example, a gradient of solute can result as a product of a chemical reaction occurring on a catalytic particle with an asymmetric distribution of catalysts (e.g., in a Janus geometry). In diffusiophoresis, the surface of the particle interacts with solute particles in a nanometer-thin boundary layer surrounding the particle [12,13]. Outside the interfacial layer, the interactions between solute particles and the surface vanish. When a self-generated concentration gradient of solute builds up as a product of a chemical reaction (e.g., in the vicinity of a Janus particle with two distinct hemispheres), a pressure gradient results at the interfacial layer. This pressure gradient induces a fluid flow causing a slip velocity around the particle at the outer edge of the boundary layer, as illustrated in Figure 1a. Phoretic effects are more prominently seen on solid particles like Janus microswimmers [6], but they also have been studied in soft vesicles [14].

### 2.2. Marangoni Effect

The Marangoni effect comes from the inhomogeneous surface tension at liquid/liquid interfaces of symmetric vesicles and droplets in response to a chemical or temperature gradients in the surrounding medium. Addition of surfactants at the interface decreases the surface tension. In the absence of a gradient, the distribution of surfactant molecules in a symmetric vesicle is homogeneous. As the concentration of surfactant molecules at the interface increases (e.g., due to a chemical reaction), the local surface tension decreases. As a result, the symmetry of the colloid is broken and active flows are induced (from low to high surface tension areas) leading to self-propulsion (Figure 1b). The Marangoni effect is most evident in the tear of wine effect, due to a concentration gradient generated by different evaporation rates of ethanol and water. Surfactant-laden active droplets, discussed further in Section 4, are paradigmatic examples of self-propulsion by Marangoni flows.

## 3. Catalytically Self-Propelled Active Vesicles

### 3.1. Liposomes

Naturally occurring amphiphiles, like phospholipids, or synthetic versions assemble into vesicles known as liposomes. These vesicles consist of a spherical lipid bilayer that resembles the lipid matrix of the cell membrane. As such, lipid bilayer membranes constitute a natural choice for the encapsulation of artificial cells, albeit far from the complexity of a biological cell membrane. The ease with which lipids self-assemble into membranous compartments has made liposomes a subject of extensive research, particularly as biocompatible carriers of small molecules [4,15] and protocell models [16,17].

Liposomes can be classified by their size and lamellar properties [4], which highly depend on the preparation method. Small unilamellar vesicles (SUVs) are vesicles of 20–100 nm diameter, large unilamellar vesicles (LUVs) are 100 nm–1 μm-sized vesicles, while the term giant unilamellar vesicles (GUVs) is typically used for vesicles larger than 10 μm. A common approach to form liposomes is the lipid-film hydration method [18]. After generating lipid films on a substrate, the hydrated lipid sheets detach during agitation and form large multilamellar vesicles. Once the vesicles form, reducing the size of the vesicles is generally achieved by sonication or extrusion [19]. In addition to lipid-film hydration, a multitude of liposome production methods have been developed, which are now widely used, including electroformation [20], lipid inverted emulsification [21], and microfluidic-based methods, such as fluid jetting [22].

Liposome membranes prepared from lipids with a low gel/liquid phase transition temperature are highly dynamic and have a large lateral fluidity, making them ideal model systems for studying membrane structure and cell division [23]. Researchers have also developed liposomes as reaction compartments for cell-free gene expression [24]. Among other functions, cell-free protein synthesis [24,25,26], the production of membrane components [27,28,29], as well as DNA amplification [30] and replication [31,32] was demonstrated inside of vesicles.

Creating liposomes capable of locomotion has proved challenging. However, steps in this direction have been taken recently. Vanderlick and colleagues [33] attached motile bacteria to propel SUVs and LUVs achieving average velocities of 28 μms^−1^ and 13 μms^−1^, respectively. Larger vesicles, however, displayed only Brownian motion, agreeing with a predicted calculation of loaded bacterial propulsion at low-Reynolds number, where the Stokes’ law applies.

Breaking of symmetry in liposomes is essential for generating propulsion. This is normally achieved by creating a structural asymmetry in the liposome. Mimicking the actin polymerisation machinery that is used by the bacterium listeria for locomotion inside a host cell, Inaba et al. have reported the propulsion of giant asymmetric liposomes driven by light-induced peptide nanofibre growth on their surface [34]. Peptide-DNA conjugates connected by a photocleavable unit were asymmetrically introduced onto phase-separated asymmetric giant liposomes. UV irradiation cleaved the conjugates and released peptide units, which self-assembled into nanofibers, driving the translational movement of the liposomes. A five-fold increase in liposome velocities was reported for active liposomes.

**Figure 2:**
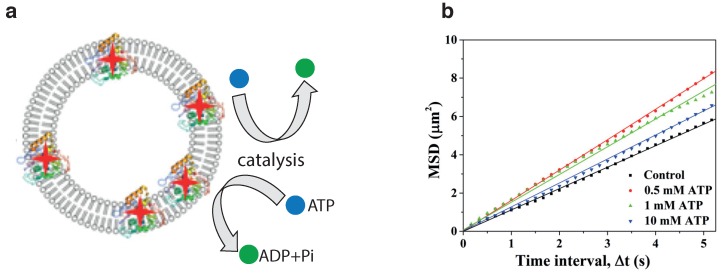
(**a**) Scheme of a liposome constructed with L-α-phosphatidylcholine (EPC) and decorated with fluorescently labelled membrane-bound ATPases catalysing the conversion of adenosine triphosphate (ATP) (in blue) to adenosine diphosphate (ADP) (in green) plus a phosphate ion (Pi); (**b**) plots showing the mean squared displacement (MSD, see Section 5.1) of ATPase tagged vesicles for different ATP concentrations as measured by optical tracking. Figures are reproduced from [35], with permission from American Chemical Society, 2019.

Encapsulation of catalysts (enzymes or nanoparticles) has not been explored as a means of phoretic propulsion for liposomes. The permeability of the liposomes to fuel molecules like H_2_O_2_ (the substrate of peroxidases like catalase) and the difficulty of generating a gradient of reaction product to produce an active flow around the vesicle pose certain challenges. An approach taken by Ghosh et al. consists of immobilizing membrane-bound enzymes onto LUVs (Figure 2a) [35]. Due to the nanoscopic size of the LUVs, the reported enhanced diffusion was assessed by Fluorescence Correlation Spectroscopy (FCS) and complemented with tracking measurements in optical microscopy. The resulting maximum enhanced diffusivity using membrane-bound ATPases was about 23% (Figure 2b) at an ATP concentration of 0.5 mM; however, it was slower in 10 mM ATP. The diffusivity of unbound fluorescent ATPases was shown to increase during substrate turnover in [35]. It is interesting to note that there is no asymmetry in these vesicles, which casts some doubt as to what the propulsion mechanism is and whether the spatial distribution of the enzymes plays a role in the vesicle motility.

### 3.2. Polymersomes

Polymersomes are vesicles formed by the self-assembly of amphiphilic synthetic block copolymers (comprising hydrophilic and hydrophobic homopolymer subunits). Polymersome-based compartments hold great promise as drug and gene delivery vehicles [36] and can be designed to respond to specific stimuli [37] (e.g., pH [38], oxidation [39], temperature [40], and light [41]) to release their cargo on demand.

Polymersomes form a hydrophobic bilayer membrane and hydrophilic core resembling liposomes. They are more robust, more malleable, and more stable vesicles compared with liposomes and show higher tissue penetration. The membrane thickness of polymersomes is typically thicker than that of liposomes, ranging from 5 to 50 nm [42], and can be engineered by changing the molecular weight of the copolymer hydrophobic block [43]. Due to chain entanglement and lateral diffusivity, the properties of polymersomes can vary depending on the polymer used and method of preparation.

Polymersomes can be made by various methods, involving either solvent-free techniques, where amphiphiles are hydrated in an aqueous medium (e.g., film rehydration, electroformation) or solvent displacement methods, where amphiphiles are dissolved in an organic solvent first followed by its removal (e.g., emulsion phase transfer, solvent injection, microfluidics) [4].

**Figure 3:**
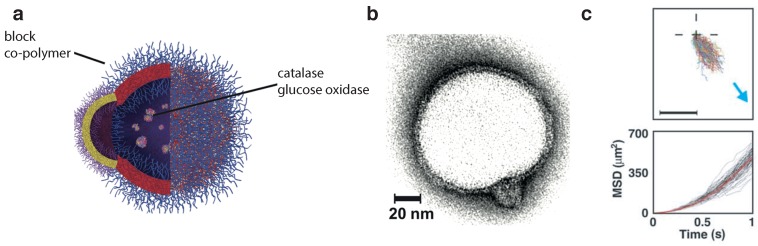
(**a**) Scheme of an asymmetric polymersome made by using a combination block co-polymers (poly[oligo(ethylene glycol) methyl methacrylate] (POEGMA)-PDPA with poly(ethylene oxide) poly(butylene oxide) (PEO-PBO)). The polymersome encapsulates glucose oxidase and catalase; (**b**) asymmetric polymersome imaged in Transmission Electron Microscopy (TEM); (**c**) normalized 1s-trajectories and corresponding mean-squared displacement (MSD for asymmetric polymersomes loaded with glucose oxidase and catalase. The arrow represents a glucose gradient. Scale bar 20 μm. Figures have been reproduced from [8], with permission from the American Association for the Advancement of Science, 2017.

Some researchers have endowed polymersomes with self-propulsion [8,9]. Such active vesicles are propelled by self-generated chemical gradients that result from asymmetric catalytic reactions occurring in the lumen of the vesicle. Joseph et al. created catalytic asymmetric polymersomes (Figure 3a,b) by encapsulating glucose oxidase and catalase in polymersomes made out of two distinct copolymers that phase-separated into two distinct domains [8]. The encapsulated enzymes work in tandem transforming glucose to D-glucono-d-lactone and water, without the formation of potentially toxic reactive oxygen species (ROS) such as hydrogen peroxide and gaseous oxygen. The resulting self-propelled polymersomes of 50 nm radius exhibit taxis within glucose gradients (Figure 3c) and have even been used in complex biological environments, notably enabling a four-fold increase in penetration of the blood brain-barrier compared with passive vesicles.

### 3.3. Stomatocytes

Apart from forming spherical vesicles, polymersomes can be manipulated to assemble into new shapes [44]. In natural cells, conformational changes are normally associated with certain diseases. As an example, human red-blood cells form a biconcave discoid shape (discocyte) under healthy conditions, but blood diseases like malaria or sickle-cell anaemia alter their shape. To induce shape changes in synthetic cells, one approach is to induce an osmotic shock by means of an imbalance in osmolarity between the inner and outer media.

**Figure 4:**
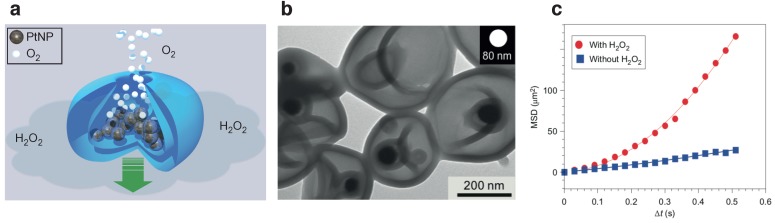
(**a**) Stomatocytes formed by the self-assembly of block copolymers polyethylene glycol-polystyrene (PEG-PS). Self-propulsion is achieved by entrapping catalytically active nanoparticles (grey, PtNPs) and immersing them in fuel (H_2_O_2_). (**b**) TEM images show the entrapment of 90 nm PtNPs in stomatocytes; (**c**) average MSD of the platinum-filled stomatocytes before and after the addition of H_2_O_2_. Figures have been reproduced from [9], with permission from Springer Nature, 2012.

Wilson et al. pioneered the fabrication of polymersomes shaped as stomatocytes (bowl-shaped erithrocytes) [9]. By kinetic manipulation of the hydrophobic portion via osmotic shock, they were able to control the deformation of these polymer vesicles made of polystyrene-block-poly(ethylene glycol) (PS-b-PEG) block copolymer. To introduce self-propulsion, Wilson and colleagues shaped the stomatocytes with a nanocavity, where catalytic particles (Pt NP) could be entrapped and an opening out of which the exhaust (water and O_2_) was released (Figure 4a,b).

This design resulted in a miniature nanoreactor of sizes ranging from 130 nm to 180 nm that could catalyze the decomposition of H_2_O_2_ by permeating inside the vesicle lumen and self-propel by an asymmetric release of oxygen bubbles through a cavity. The active Brownian motion of the stomatocytes, as measured by Nanoparticle Tracking Analysis (NTA), resulted in an average ballistic velocity of 23 μms^−1^ (Figure 4c), albeit randomized by rotational Brownian motion.

Active stomatocytes have also been implemented with enzymes, such as glucose oxidase and catalase [45] and other enzymatic cascades [46]. Stomatocytes reached three times higher velocities than those encapsulated with Pt nanoparticles. In addition, stomatocytes with temperature-dependent velocities have been reported, which make use of temperature-responsive polymer brushes to hinder permeation of fuel through the membrane [47]. Most of these self-propelled structures perform ballistic motion but are randomized by thermal forces, giving rise to a persistent random walk. This manifests itself in an enhanced effective diffusion constant at larger time scales than the rotational diffusion time. Controlled directional motility was achieved by encapsulating Pt-Ni nanoparticles, which rendered stomatocytes both catalytically active and magnetic [48]. More recently, catalase encapsulated stomatocytes of even smaller sizes have been reported capable of crossing the blood–brain barrier [49].

### 3.4. Other Protocell Models

A new type of model protocell based on the self-assembly of DNA and clay was developed by Kumar et al. [50]. The organoclay/DNA membrane-less capsules were fabricated by complexation at the surface of DNA/enzyme droplets in a dispersion of organoclay sheets. Two different types of enzymes—catalase and glucose oxidase—were trapped inside organoclay/DNA microcapsules and were used as chemical engines to switch on or turn-off, respectively, the formation of oxygen bubbles. The accumulation and periodic consumption of gas bubbles resulted in an oscillatory motion of buoyant microcapsules up and down the water column, mimicking the locomotion of certain cyanobacteria.

An amino acid derived supramolecular hydrogel was used to produce a protocell model with a primitive cytoskeleton-like interior. These vesicles underwent chemically driven self-propulsion when Pt nanoparticles were attached to their external surface asymmetrically and H_2_O_2_ was added to the solution [51]. Emerging systems such as proteinosomes and colloidosomes have also been considered as possible artificial cell models, which could potentially be propelled by the methods discussed above.

## 4. Active Droplets

There have been several examples of droplets capable of locomotion, i.e., active droplets. While such liquid droplets cannot yet be regarded as cell-like, due to their propensity to coalesce into larger structures and the absence of a membrane, they are useful models as chemical reactors. These are normally emulsion droplets that can be produced by simply dropping the dispersed phase from a pipette or a nozzle into the continuous phase. Typical examples of emulsions include oil-in-water or water-in-oil droplets. Stability and prevention of coalescence between droplets are achieved by the addition of surfactants that lower the interfacial tension. Large-scale production of droplets can be achieved by shaking and sonication. The use of microfluidic devices allows the creation of monodisperse and more complex droplet architectures (e.g., double emulsions) [52].

The overarching propulsion mechanism for active droplets is due to gradients in the surface tension along the interface of the droplet. These can be generated by either an external field or by the droplet itself. The former involves changing the wettability of the substrate. For example, Chaudhury and Whitesides first showed the spontaneous motion of a droplet moving uphill, generated by a hydrophobicity gradient on a substrate [53]. In electrowetting-on dielectric (EWOD), droplets placed on a hydrophobic layer move by changes in the surface-tension forces induced by an external electric field [54]. Tuneable wettability of the substrate allows for complex droplet motion such as transport, division, and merging.

**Figure 5:**
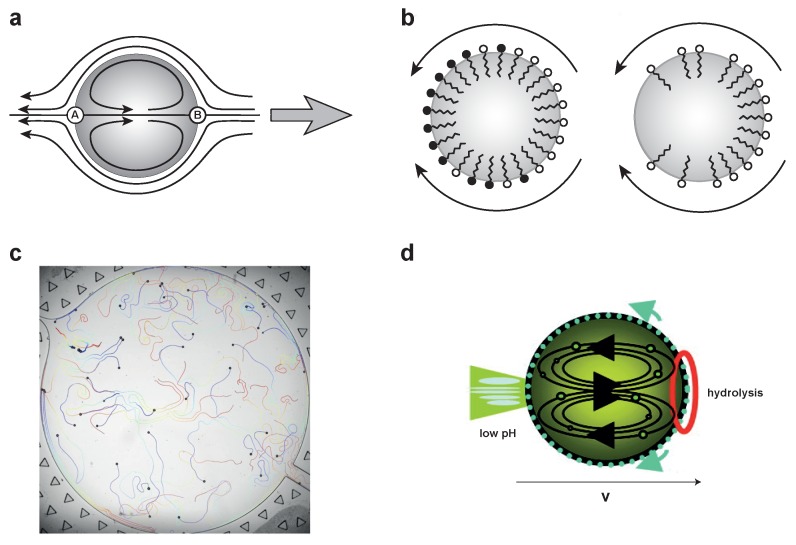
(**a**) Flow fields around and inside a moving droplet. The scheme shows the formation of a convection roll inside the droplet; (**b**) mechanisms to generate Marangoni stresses on droplet/aqueous phase interface: (left) surfactant molecules undergo a chemical reaction, thereby changing the surface tension; (right) the surfactant density is inhomogeneous created by solubilization. The grey arrows point towards the region with higher surface tension. The droplet propels in the opposite direction; (**c**) Spontaneous motion consisting of pure water droplets in an oil-surfactant medium of squalane and monoolein as described in [55]. (**d**) spontaneous motion of an oil droplet in an aqueous surfactant solution by phase separation, as shown in [56]. The hydrolyzed surfactant moves from the leading edge to the anterior pole. (**a**,**b**) and have been adapted from [61,62], with permission from Royal Society of Chemistry, 2014; (**c**) from [55], American Physical Society, 2014, and (**d**) has been reproduced from [56], American Chemical Society, 2007.

Surface tension gradients at liquid/liquid interfaces lead to Marangoni flows (Figure 5a). In active droplets, such gradients can be sustained by chemical reactions, which affect the surfactant molecules, thereby changing the surface tension at the interface of the droplet (Figure 5b left). A second self-propulsion mechanism leading to Marangoni flows involves the inhomogeneous surfactant concentration along the droplet created during a process of solubilization (Figure 5b right). In this mechanism, droplet oil molecules, together with surfactants on the surface, dissolve into micelles in the aqueous phase creating a spatially inhomogeneous surfactant concentration. An example of propulsion by solubilization is shown in Figure 5c, where water droplets self-propel in an oil-surfactant medium of squalane and monoolein [55]. Both mechanisms create a gradient in surface tension along the interface. This gradient has dimensions of stress and is balanced by viscous stresses in the liquid, causing a viscous shear flow leading to droplet propulsion.

An alternative mechanism owes self-propulsion to the phase separation dynamics driven by liquid–liquid phase transitions [56]. An initially homogeneous surfactant layer spontaneously breaks symmetry by hydrolysis of the surfactant precursor. This occurs at the leading edge of the droplet while the hydrolyzed surfactant moves to the anterior pole, leading to internal convection, a self-generated pH gradient, and self-propulsion as shown in Figure 5d.

A characteristic reference velocity for the motion of active droplets in the fluid may be obtained from the tangential stress balance at the interface of the drop $v∼\frac{R\nabla \gamma }{\eta }$ [57,58]. For active droplets of radius R = 50 μm, a difference in surface tension of ∇γ = $10^{-3}$ N/m and viscosities of η on the order of 1 mPa · s yields propulsion velocities of the order of 50 μms^−1^. Reported typical propulsion velocities are of the order of 10 μms^−1^ and the cruising ranges are about 100–4000 droplet diameters [58].

An interesting research avenue in active matter has emerged that studies the collective motion of large populations of active droplets. Dense ensembles can be easily fabricated with microfluidic tools and give rise to hydrodynamic interactions that result in collective effects like clustering and chemotaxis. Active droplets have exhibited chemotactic behavior towards sodium chloride [59] and low pH regions, and have been shown to navigate through a centimeter-scale maze to seek a source of acid [60].

## 5. Measuring Self-Propelled Vesicles and Droplets

The experimental tools to characterize the motility of vesicles differ from those used to study their structure (e.g., chromatography, TEM, or confocal microscopy). Self-propulsion is manifested in the form of an enhanced diffusivity caused by a combination of ballistic propulsion and rotational Brownian motion. Two general measurement approaches can be distinguished: on the one hand, single-particle methods track individual particles and extract their propulsion velocity by measuring average displacements. On the other, ensemble measurement methods can probe the diffusion coefficient of self-propelled structures. Experimental methods used for measuring self-propelled vesicles are described below:

### 5.1. Optical Microscopy

Optical microscopy is the most commonly used method to characterize the motility of active particles. This approach involves imaging and tracking trajectories of individual particles. This results in a list of coordinates as a function of time that can then be used to calculate the mean squared displacement, as an average of *n* squared-displacements corresponding to a given time step (τ):

(1) $$MSD=<L^{2}>\left(\tau \right)=\frac{1}{n}\sum_{i=1}^{n}\Delta r_{i}^{2}\left(\tau \right).$$

For the case of particles diffusing in 2D, $<L^{2}{>}_{diffusive}$ = 4Dτ holds for passive particles, while ballistic behavior would be characterized by $<L^{2}{>}_{ballistic}$ = $v^{2}\tau^{2}$. Active particles show a combination of both regimes which contribute to a characteristic functional form of the MSD [6]. The rotational diffusion time sets the timescale that separates these two regimes. At times longer than the rotational diffusion time, rotational diffusion leads to a randomization of the direction of propulsion, and the particle undergoes a random walk whose step length is the product of the propelled velocity *v* and the rotational diffusion time $\tau_{D}$, leading to an enhancement of the effective diffusion coefficient.

Tracking methods with optical microscopy are suitable for vesicles larger than the resolution limit set by the diffraction limit. Self-propelled LUVs and GUVs have been tracked with fluorescence microscopy [35]. Droplets are observed mostly via standard 2D video microscopy. Further information can be extracted by using polarization filters (for liquid crystal droplets) to probe nematic ordering. Addition of small tracing colloidal particles can be used to map flow fields via Particle Image Velocimetry (PIV).

### 5.2. Dynamic Light Scattering

Dynamic light scattering (DLS) is useful to measure the enhanced diffusion coefficient of an ensemble of monodisperse nano/micro motors that scatter light strongly. This technique has been used to study the propulsion of stomatocytes [9]. In this method, a coherent light source (e.g., laser) passes through a sample and the scattered light is recorded by a photodiode at a fixed angle. Fluctuations in the scattered light are processed by a digital correlator to produce a correlogram out of which a diffusion coefficient is extracted. Self-propelled particles appear smaller than their real size when measuring the hydrodynamic radius.

### 5.3. Nanoparticle Tracking Analysis

Nanoparticle tracking analysis (NTA) provides a tracking method for measuring the diffusion and concentration of particles from about 30 nm to 1000 nm, with the lower detection limit being dependent on the dielectric properties of the nanoparticles. The technique consists of a laser scattering setup similar to DLS but with a custom-made microfluidic device and a charge-coupled device (CCD) camera, which permits the visualization of nanoparticles by recording their scattered light. A laser beam is passed through the sample chamber, and the particles in suspension in the path of the beam scatter light and are visualized on the camera. The software is able to identify the position, track, and measure the average displacement within a fixed time frame. The particle size is derived from the Einstein–Stokes equation; therefore self-propelled particles exhibit a smaller ”apparent” size due to their enhanced diffusivity. NTA provides individual particle-by-particle analysis rather than an ensemble measurement. Compared with DLS, nanoparticle tracking considers individual particles and provides a higher resolution for multimodal particles. NTA can measure the signal from single particles, but it has a fundamental limitation due to the low scattering of small particles. NTA may also be used to track fluorescent particles by detecting the fluorescence signal rather than scattered light by modifying the optical setup. A similar direct single-molecule imaging approach has been used to observe the diffusion of individual enzymes in solution [63]. NTA has been used to study the propulsion of Pt loaded stomatocytes [9].

### 5.4. Fluorescence Correlation Spectroscopy

Fluorescence Correlation Spectroscopy (FCS) is a single-molecule method that measures fluctuations in fluorescence intensity as a result of particles diffusing in and out of a diffraction-limited confocal volume. Single molecules in this volume of excitation (usually around 1 fL) are sufficient to produce a signal. FCS data analysis uses correlation functions to extract the diffusion constant from a fluctuating signal, similar to those used in Dynamic Light Scattering. The advantage of FCS lies in its selectivity, as only the motion of fluorescent particles is detected, while unlabelled impurities remain undetected. A plethora of fluorescently labelled enzymes has been studied with FCS [64,65] and Stimulated Emission Depletion (STED) FCS [66]. Some studies have suggested that photophysical artifacts and enzyme dissociation can perturb FCS measurements [67]. Recently, the enhanced diffusivity of fluorescently labelled LUVs functionalized with membrane-bound proteins has been reported using FCS [35].

### 5.5. Alternative Methods

Beyond scattering and fluorescence techniques, alternative methods have been utilized to study the active propulsion and enhanced diffusivity of nanoscopic structures. Pulsed Field Gradient Nuclear Magnetic Resonance (PFG-NMR) has been used to study the enhanced diffusivity of active enzymes [68] and molecules [69]. The use of PFG-NMR to characterize the size distribution for polymersomes [70] suggests this method could also be used to measure the enhanced diffusivity of nanoscopic vesicles.

Electrochemical methods have also been introduced recently as measurement tools for self-propelled nanoparticles [71] and microparticles [72]. Nano-impact event frequency on microelectrodes provides a proxy for the diffusivity of colliding active particles. Nanoimpact voltammetry experiments with vesicles [73] indicate that this approach could be implemented with self-propelled vesicles and droplets.

## 6. Conclusions and Perspective

Table 1 summarizes different experimental realizations of motile vesicles and droplets. They vary in sizes, compositions, propulsion mechanisms, and observation methods. Thus far, no system has achieved a true level of autonomy and control and the mechanisms driving their motion are still not well understood. The nanometer-sized vesicles (liposomes and polymersomes) are small compared with natural cell compartments. Active droplets, in contrast, achieve measurable velocities and are as large as real cells. Nevertheless, these first steps towards motile artificial cells are valuable and set the stage towards further research at the interface of active matter and synthetic biology.

The attraction towards or away from chemicals is a feature of locomotion in cells, known as chemotaxis. Already primitive forms of chemotaxis are possible with self-propelled asymmetric polymersomes and active droplets, as discussed in this review. However, a natural chemotactic machinery such as the one found in *E. coli*, apart from motility, it equips bacteria with sensing, memory, and signaling abilities that allow them to migrate towards favorable environments and away from unfavorable ones. Implementing analogous chemotaxis in artificial cells will require coupling of propulsion mechanisms with more complex and adaptive chemical reaction networks.

In the long run, such chemotactic vehicles could potentially be used as therapeutic agents that actively target diseased cells and tissues (as Ehrlich’s “magic bullet”). The capabilities of motile containers can further be expanded by functionalization with DNA or peptide-based nanostructures, which could provide mechanisms for binding, sensing, or controlled fusion with cells or other compartments. Such chemotactic and adaptive artificial cells capable of sensing and responding to their environment may also be regarded as “soft robots” at the cellular scale.

Motile cell-scale structures are also of considerable interest in the context of origin of life research. The problem of the origin of life has been approached by different schools of thought from different angles, in particular with respect to the roles of metabolism vs. replication at the onset of life [74]. A general consensus combines both into an “information-compartment-metabolism first” hypothesis. Furthermore, a “movement-first” hypothesis advocates that an early capacity for adaptive self-motility was already present at the origin of life [75]. Motile artificial compartments enable the exploration of transport phenomena that might have played a role in prebiotic conditions.

## Figures and Tables

**Table 1:** Propulsion strategies for vesicles and droplets, observation methods and reported average velocities or changes in diffusion coefficients.

Compartment	Size	Propulsion Strategy	Observation Method	Velocity/$\frac{\Delta D}{D_{0}}$	Reference
Liposomes	100 nm	Phoresis (enzymes)	FCS, Optical tracking	$\frac{\Delta D}{D_{0}}∼$23–35%	[35]
Polymersomes	100 nm	Phoresis (enzymes)	NTA	200 μms^−1^	[8]
Stomatocytes	150 nm	Phoresis (Pt NPs)	NTA, DLS	23 μms^−1^	[9]
Droplets	20–60 μm	Marangoni flows	Optical tracking	10–50 μms^−1^	[55]

## References

[1] Jia H., Schwille P. (2019). Bottom-up synthetic biology: Reconstitution in space and time. Curr. Opin. Biotechnol..

[2] Villar G., Graham A.D., Bayley H. (2013). A tissue-like printed material. Science.

[3] Dzieciol A.J., Mann S. (2012). Designs for life: Protocell models in the laboratory. Chem. Soc. Rev..

[4] Rideau E., Dimova R., Schwille P., Wurm F.R., Landfester K. (2018). Liposomes and polymersomes: A comparative review towards cell mimicking. Chem. Soc. Rev..

[5] Marchetti M.C., Joanny J.F., Ramaswamy S., Liverpool T.B., Prost J., Rao M., Simha R.A. (2013). Hydrodynamics of soft active matter. Rev. Mod. Phys..

[6] Howse J.R., Jones R.A., Ryan A.J., Gough T., Vafabakhsh R., Golestanian R. (2007). Self-motile colloidal particles: From directed propulsion to random walk. Phys. Rev. Lett..

[7] Bechinger C., Di Leonardo R., Löwen H., Reichhardt C., Volpe G., Volpe G. (2016). Active particles in complex and crowded environments. Rev. Mod. Phys..

[8] Joseph A., Contini C., Cecchin D., Nyberg S., Ruiz-Perez L., Gaitzsch J., Fullstone G., Tian X., Azizi J., Preston J. (2017). Chemotactic synthetic vesicles: Design and applications in blood-brain barrier crossing. Sci. Adv..

[9] Wilson D.A., Nolte R.J.M., van Hest J.C.M. (2012). Autonomous movement of platinum-loaded stomatocytes. Nat. Chem..

[10] Sundararajan S., Lammert P.E., Zudans A.W., Crespi V.H., Sen A. (2008). Catalytic motors for transport of colloidal cargo. Nano Lett..

[11] Anderson J.L. (1989). Colloid transport by interfacial forces. Annu. Rev. Fluid Mech..

[12] Kapral R. (2012). Nanomotors Propelled by Chemical Reactions. Eng. Chem. Complex..

[13] Golestanian R., Liverpool T.B., Ajdari A. (2005). Propulsion of a molecular machine by asymmetric distribution of reaction products. Phys. Rev. Lett..

[14] Gupta S., Sreeja K.K., Thakur S. (2015). Autonomous movement of a chemically powered vesicle. Phys. Rev. E.

[15] Lasic D. (1992). Liposomes. Am. Sci..

[16] Szostak J.W., Bartel D.P., Luisi P.L. (2001). Synthesizing life. Nature.

[17] Fenz S.F., Sengupta K. (2012). Giant vesicles as cell models. Integr. Biol..

[18] Zhang H. (2017). Thin-film hydration followed by extrusion method for liposome preparation. Liposomes.

[19] Lapinski M.M., Castro-Forero A., Greiner A.J., Ofoli R.Y., Blanchard G.J. (2007). Comparison of liposomes formed by sonication and extrusion: rotational and translational diffusion of an embedded chromophore. Langmuir.

[20] Angelova M., Dimitrov D.S. (1988). A mechanism of liposome electroformation. Trends in Colloid and Interface Science II.

[21] Pautot S., Frisken B.J., Weitz D. (2003). Production of unilamellar vesicles using an inverted emulsion. Langmuir.

[22] Stachowiak J.C., Richmond D.L., Li T.H., Liu A.P., Parekh S.H., Fletcher D.A. (2008). Unilamellar vesicle formation and encapsulation by microfluidic jetting. Proc. Natl. Acad. Sci. USA.

[23] Deshpande S., Spoelstra W.K., van Doorn M., Kerssemakers J., Dekker C. (2018). Mechanical division of cell-sized liposomes. ACS Nano.

[24] Noireaux V., Libchaber A. (2004). A vesicle bioreactor as a step toward an artificial cell assembly. Proc. Natl. Acad. Sci. USA.

[25] Maeda Y.T., Nakadai T., Shin J., Uryu K., Noireaux V., Libchaber A. (2011). Assembly of MreB filaments on liposome membranes: A synthetic biology approach. ACS Synth. Biol..

[26] Soga H., Fujii S., Yomo T., Kato Y., Watanabe H., Matsuura T. (2013). In vitro membrane protein synthesis inside cell-sized vesicles reveals the dependence of membrane protein integration on vesicle volume. ACS Synth. Biol..

[27] Scott A., Noga M.J., de Graaf P., Westerlaken I., Yildirim E., Danelon C. (2016). Cell-free phospholipid biosynthesis by gene-encoded enzymes reconstituted in liposomes. PLoS ONE.

[28] Vogele K., Frank T., Gasser L., Goetzfried M.A., Hackl M.W., Sieber S.A., Simmel F.C., Pirzer T. (2018). Towards synthetic cells using peptide-based reaction compartments. Nat. Commun..

[29] Bhattacharya A., Brea R.J., Niederholtmeyer H., Devaraj N.K. (2019). A minimal biochemical route towards de novo formation of synthetic phospholipid membranes. Nat. Commun..

[30] Oberholzer T., Albrizio M., Luisi P.L. (1995). Polymerase chain reaction in liposomes. Chem. Biol..

[31] Kita H., Matsuura T., Sunami T., Hosoda K., Ichihashi N., Tsukada K., Urabe I., Yomo T. (2008). Replication of genetic information with self-encoded replicase in liposomes. ChemBioChem.

[32] Van Nies P., Westerlaken I., Blanken D., Salas M., Mencía M., Danelon C. (2018). Self-replication of DNA by its encoded proteins in liposome-based synthetic cells. Nat. Commun..

[33] Dogra N., Izadi H., Vanderlick T.K. (2016). Micro-motors: A motile bacteria based system for liposome cargo transport. Sci. Rep..

[34] Inaba H., Uemura A., Morishita K., Kohiki T., Shigenaga A., Otaka A., Matsuura K. (2018). Light-induced propulsion of a giant liposome driven by peptide nanofibre growth. Sci. Rep..

[35] Ghosh S., Mohajerani F., Son S., Velegol D., Butler P.J., Sen A. (2019). Motility of Enzyme-Powered Vesicles. Nano Lett..

[36] Lomas H., Canton I., MacNeil S., Du J., Armes S.P., Ryan A., Lewis A., Battaglia G. (2007). Biomimetic pH Sensitive Polymersomes for Efficient DNA Encapsulation and Delivery. Adv. Mater..

[37] Hu X., Zhang Y., Xie Z., Jing X., Bellotti A., Gu Z. (2017). Stimuli-responsive polymersomes for biomedical applications. Biomacromolecules.

[38] Messager L., Gaitzsch J., Chierico L., Battaglia G. (2014). Novel aspects of encapsulation and delivery using polymersomes. Curr. Opin. Pharmacol..

[39] Scott E.A., Stano A., Gillard M., Maio-Liu A.C., Swartz M.A., Hubbell J.A. (2012). Dendritic cell activation and T cell priming with adjuvant-and antigen-loaded oxidation-sensitive polymersomes. Biomaterials.

[40] Qin S., Geng Y., Discher D.E., Yang S. (2006). Temperature-Controlled Assembly and Release from Polymer Vesicles of Poly (ethylene oxide)-block-poly (N-isopropylacrylamide). Adv. Mater..

[41] Peyret A., Ibarboure E., Tron A., Beauté L., Rust R., Sandre O., McClenaghan N.D., Lecommandoux S. (2017). Polymersome Popping by Light-Induced Osmotic Shock under Temporal, Spatial, and Spectral Control. Angew. Chem. Int. Ed..

[42] Chandrawati R., Caruso F. (2012). Biomimetic liposome-and polymersome-based multicompartmentalized assemblies. Langmuir.

[43] Smart T., Lomas H., Massignani M., Flores-Merino M.V., Perez L.R., Battaglia G. (2008). Block copolymer nanostructures. Nano Today.

[44] Salva R., Meins J.F.L., Sandre O., Brûlet A., Schmutz M., Guenoun P., Lecommandoux S. (2013). Polymersome shape transformation at the nanoscale. ACS Nano.

[45] Abdelmohsen L.K.E.A., Nijemeisland M., Pawar G.M., Janssen G.J.A., Nolte R.J.M., van Hest J.C.M., Wilson D.A. (2016). Dynamic Loading and Unloading of Proteins in Polymeric Stomatocytes: Formation of an Enzyme-Loaded Supramolecular Nanomotor. ACS Nano.

[46] Nijemeisland M., Abdelmohsen L.K.E.A., Huck W.T.S., Wilson D.A., van Hest J.C.M. (2016). A Compartmentalized Out-of-Equilibrium Enzymatic Reaction Network for Sustained Autonomous Movement. ACS Cent. Sci..

[47] Tu Y., Peng F., Sui X., Men Y., White P.B., van Hest J.C., Wilson D.A. (2017). Self-propelled supramolecular nanomotors with temperature-responsive speed regulation. Nat. Chem..

[48] Peng F., Tu Y., Men Y., van Hest J.C., Wilson D.A. (2017). Supramolecular adaptive nanomotors with magnetotaxis behavior. Adv. Mater..

[49] Sun J., Mathesh M., Li W., Wilson D.A. (2019). Enzyme-Powered Nanomotors with Controlled Size for Biomedical Applications. ACS Nano.

[50] Kumar B.V.V.S.P., Patil A.J., Mann S. (2018). Enzyme-powered motility in buoyant organoclay/DNA protocells. Nat. Chem..

[51] Krishna Kumar R., Yu X., Patil A.J., Li M., Mann S. (2011). Cytoskeletal-like Supramolecular Assembly and Nanoparticle-Based Motors in a Model Protocell. Angew. Chem. Int. Ed..

[52] Chu L.Y., Utada A.S., Shah R.K., Kim J.W., Weitz D.A. (2007). Controllable monodisperse multiple emulsions. Angew. Chem. Int. Ed..

[53] Chaudhury M.K., Whitesides G.M. (1992). How to make water run uphill. Science.

[54] Pollack M.G., Fair R.B., Shenderov A.D. (2000). Electrowetting-based actuation of liquid droplets for microfluidic applications. Appl. Phys. Lett..

[55] Izri Z., van der Linden M.N., Michelin S., Dauchot O. (2014). Self-Propulsion of Pure Water Droplets by Spontaneous Marangoni-Stress-Driven Motion. Phys. Rev. Lett..

[56] Hanczyc M.M., Toyota T., Ikegami T., Packard N., Sugawara T. (2007). Fatty acid chemistry at the oil-water interface: Self-propelled oil droplets. J. Am. Chem. Soc..

[57] Balasubramaniam R., Subramanian R.S. (2004). Thermocapillary Migration of a Drop. Ann. N. Y. Acad. Sci..

[58] Seemann R., Fleury J.B., Maass C.C. (2016). Self-propelled droplets. Eur. Phys. J. Spec. Top..

[59] Cejkova J., Novak M., Stepanek F., Hanczyc M.M. (2014). Dynamics of chemotactic droplets in salt concentration gradients. Langmuir.

[60] Lagzi I., Soh S., Wesson P.J., Browne K.P., Grzybowski B.A. (2010). Maze solving by chemotactic droplets. J. Am. Chem. Soc..

[61] Herminghaus S., Maass C., Krüger C., Thutupalli S., Goehring L., Bahr C. (2014). Interfacial mechanisms in active emulsions. Soft Matter.

[62] Maass C.C., Krüger C., Herminghaus S., Bahr C. (2016). Swimming droplets. Annu. Rev. Condens. Matter Phys..

[63] Xu M., Ross J.L., Valdez L., Sen A. (2019). Direct Single Molecule Imaging of Enhanced Enzyme Diffusion. Phys. Rev. Lett..

[64] Riedel C., Gabizon R., Wilson C.A., Hamadani K., Tsekouras K., Marqusee S., Presse S., Bustamante C. (2015). The heat released during catalytic turnover enhances the diffusion of an enzyme. Nature.

[65] Sen S., Dey K.K., Muddana H.S., Tabouillot T., Ibele M.E., Butler P.J., Sen A. (2013). Enzyme molecules as nanomotors. J. Am. Chem. Soc..

[66] Jee A.Y., Cho Y.K., Granick S., Tlusty T. (2018). Catalytic enzymes are active matter. Proc. Natl. Acad. Sci. USA.

[67] Gunther J.P., Borsch M., Fischer P. (2018). Diffusion measurements of swimming enzymes with fluorescence correlation spectroscopy. Accounts Chem. Res..

[68] Guenther J.P., Majer G., Fischer P. (2019). Absolute diffusion measurements of active enzyme solutions by NMR. J. Chem. Phys..

[69] Pavlick R.A., Dey K.K., Sirjoosingh A., Benesi A., Sen A. (2013). A catalytically driven organometallic molecular motor. Nanoscale.

[70] Valentini M., Vaccaro A., Rehor A., Napoli A., Hubbell J.A., Tirelli N. (2004). Diffusion NMR spectroscopy for the characterization of the size and interactions of colloidal matter: The case of vesicles and nanoparticles. J. Am. Chem. Soc..

[71] Jiang L., Santiago I., Foord J. (2017). Observation of nanoimpact events of catalase on diamond ultramicroelectrodes by direct electron transfer. Chem. Commun..

[72] Moo J.G.S., Pumera M. (2016). Self-propelled micromotors monitored by particle-electrode impact voltammetry. ACS Sens..

[73] Toh H., Compton R. (2015). Electrochemical detection of single micelles through ‘nano-impacts’. Chem. Sci..

[74] Anet F.A. (2004). The place of metabolism in the origin of life. Curr. Opin. Chem. Biol..

[75] Froese T., Virgo N., Ikegami T. (2014). Motility at the origin of life: Its characterization and a model. Artif. Life.

